# FGF23 Controls Myocardial Fibrosis Progression via Promoting Cardiac Fibroblast Proliferation and Activation in Mice

**DOI:** 10.3390/biology15070539

**Published:** 2026-03-27

**Authors:** Leyi Shen, Mingqi Hu, Mei Xue, Santie Li

**Affiliations:** School of Pharmaceutical Science, Wenzhou Medical University, Wenzhou 325035, China; sly1212320020804@163.com (L.S.); mingqihu01@163.com (M.H.); wzmuxm@163.com (M.X.)

**Keywords:** cardiac fibrosis, cardiac fibroblasts, FGF23, FGFR4

## Abstract

Heart failure (HF) is the leading cause of morbidity and mortality worldwide, while myocardial fibrosis acts as a pivotal hallmark, which exacerbates ventricular dysfunction and remodeling in HF. In this study, we found FGF23, a critical endocrine regulator, which regulates phosphate and vitamin D metabolism, was significantly upregulated in fibrotic mouse hearts after transverse aortic constriction (TAC). By using the FGF23 monoclonal antibody, we found that inhibition of FGF23 alleviated TAC-induced cardiac fibrosis, while injection of recombinant FGF23 (rFGF23) protein exacerbated tissue fibrosis in mouse hearts after TAC. RNA sequencing indicated that FGF23 may promote cardiac fibroblast proliferation and activation in stressed mouse hearts. In human primary cardiac fibroblasts, rFGF23 treatment further upregulated the expression of Ki67, Cyclin D1, Cyclin E1, PCNA, α-SMA, and collagen 1A1 after TGF-β stimulation. Further results indicated that FGF23 promoted cardiac fibroblast proliferation and activation through FGFR4 and activated the downstream MAPK/ERK signaling. This study suggests a role of FGF23 in the regulation of myocardial fibrosis, which shows the potential of targeting FGF23 in the treatment of HF and cardiac fibrosis.

## Simple Summary

Heart failure remains a leading cause of illness and death worldwide, and a key feature worsening this condition is scarring of the heart muscle, clinically termed myocardial fibrosis. In the current investigation, we identified a hormone, FGF23, which is usually involved in regulating minerals like phosphate. We found that the level of FGF23 was increased in mouse hearts experiencing fibrosis after a procedure that mimics heart failure. When we blocked FGF23, the heart scarring decreased. Conversely, adding FGF23 made the scarring worse. Our research suggests that FGF23 promotes the growth and activation of cells responsible for scar tissue formation in stressed hearts. This discovery highlights FGF23 as a potential target for developing new treatments for heart failure and its associated scarring, offering hope for improving patient outcomes.

## 1. Introduction

Heart failure (HF) represents a major global health burden with high rates of hospitalization and mortality [1,2]. A central pathological feature driving HF progression is myocardial fibrosis, characterized by the excessive deposition of extracellular matrix (ECM) proteins, primarily collagens, by activated cardiac fibroblasts [3,4]. This process leads to increased myocardial stiffness, impaired diastolic and systolic function, electrical conduction abnormalities, and ultimately, adverse ventricular remodeling [4,5]. Despite advances in HF therapy targeting neurohormonal axes, effective anti-fibrotic strategies remain an unmet clinical need, underscoring the importance of identifying potential molecular regulators of cardiac fibrosis.

Fibroblast growth factor 23 (FGF23) is a hormone primarily known for its role in regulating phosphate and vitamin D metabolism [6,7]. Previous studies suggest that FGF23 is produced mainly by osteocytes and osteoblasts, and its actions are mediated through FGF receptors (FGFRs) and the co-receptor α-klotho [7,8]. While FGF23’s classical functions are well-established in mineral homeostasis, emerging evidence suggests its involvement in a broader range of physiological and pathological processes, including cardiovascular diseases [9,10]. Meanwhile, studies have indicated that elevated FGF23 levels are associated with adverse cardiovascular outcomes, including hypertension, left ventricular hypertrophy, and HF [11,12]. FGF23 and its receptor FGFR4 were shown to have the ability to induce cardiomyocyte hypertrophy in disease conditions [10,11,12]. However, the precise mechanisms by which FGF23 contributes to cardiac fibrosis, particularly in pressure overload, are not fully understood.

Upon exposure to pathological mechanical or biochemical stimuli, quiescent resident cardiac fibroblasts experience a dramatic phenotypic transition, differentiating into highly active myofibroblasts [13]. These newly formed myofibroblasts are characterized by their augmented proliferative rates and unparalleled proficiency in facilitating tissue contraction and ECM scar formation [14,15]. The activation of these myofibroblasts constitutes the defining watershed between beneficial adaptive tissue repair and highly maladaptive, irreversible fibrosis [16,17]. Consequently, deciphering the precise molecular triggers that govern the cell cycle re-entry and phenotypic shift of cardiac fibroblasts is important for the design of targeted anti-fibrotic therapeutics.

In this study, we found that FGF23 expression was upregulated in pressure-overloaded mouse hearts. Using gain- and loss-of-function approaches in vivo, we demonstrated that FGF23 exacerbates TAC-induced cardiac fibrosis. Furthermore, through RNA sequencing and in vitro experiments with human primary cardiac fibroblasts, we identified that FGF23 drives cardiac fibroblast proliferation and activation via FGFR4 and the mitogen-activated protein kinase (MAPK)/ERK signaling pathway. This study provides compelling evidence for a role of FGF23 in promoting cardiac fibrosis by enhancing cardiac fibroblast proliferation and activation, highlights that FGF23 can serve as a potential therapeutic target for managing myocardial fibrosis, and targeting FGF23 may improve outcomes in patients with heart failure.

## 2. Materials and Methods

### 2.1. Animals

All in vivo experimental protocols and animal handling procedures were formally reviewed and approved by the Institutional Animal Care and Research Advisory Committee of Wenzhou Medical University. All studies strictly adhered to the comprehensive guidelines set forth by the National Institutes of Health Guide for the Care and Use of Laboratory Animals. Male C57BL/6J mice (aged 8 to 10 weeks) were purchased from Vital River Laboratory Animal Technology Co., Ltd. (Beijing, China). The murine subjects were maintained in a highly controlled, specific-pathogen-free environment, featuring a standardized 12-h light/dark circadian cycle, with unrestricted ad libitum access to regular laboratory rodent chow and sterilized drinking water.

### 2.2. Mouse Model of Myocardial Fibrosis

To experimentally induce progressive myocardial fibrosis, the well-characterized Transverse Aortic Constriction (TAC) surgical model was implemented. The 8- to 10-week-old male C57BL/6J mice were randomly allocated into specific experimental cohorts (Sham vs. TAC). The surgical procedure commenced with profound anesthesia induced via isoflurane inhalation. Subsequently, a minimally invasive left thoracotomy was performed at the second intercostal space to meticulously expose the aortic arch. Constriction of the transverse aorta was achieved by tightly ligating a 7-0 nylon suture around the vessel against a customized 27-gauge blunt needle. Immediate withdrawal of the needle yielded a highly reproducible and standardized luminal stenosis of approximately 0.4 mm in diameter. Animals designated for the sham-operated control group underwent an identical surgical exposure, including isolation of the aorta, but explicitly lacked the application of the constricting ligature. Following a postoperative recovery period of eight weeks, the animals were humanely euthanized to harvest cardiac tissues for subsequent downstream molecular and histological evaluations.

For FGF23 monoclonal antibody (FGF23 mAb, #HY-P9939; MCE, Monmouth Junction, NJ, USA) treatment, 16 mg/kg FGF23 mAb was subcutaneously injected once a week after TAC operation [18]. Recombinant FGF23 (rFGF23) was purchased from Ipodix Biotechnology Engineering Co., Ltd. (#PA1000-8189; Wuhan, China) and was subcutaneously injected every two days after TAC operation at the dose of 0.5 mg/kg [19].

### 2.3. Human Primary Cardiac Fibroblasts

Human primary cardiac fibroblasts were commercially acquired from Procell (#CP-H078; Wuhan, China), and cells were passaged and seeded onto culture dishes pre-coated with laminin to optimize adherence. The in vitro maintenance medium consisted of high-glucose Dulbecco’s Modification of Eagle’s Medium (DMEM) (#11995065; Gibco, Grand Island, NY, USA) supplemented with 2% fetal bovine serum (#A5670801; Gibco, Grand Island, NY, USA) and 1% penicillin/streptomycin (#15140122; Gibco, Grand Island, NY, USA). The cell cultures were continuously incubated in a humidified incubator with 5% CO_2_ at 37 °C.

TGF-β (20 ng/mL) was added with or without rFGF23 (50 ng/mL) in the culture medium of human primary cardiac fibroblasts for 48 h.

For RNA interference, human primary cardiac fibroblasts were transfected with control scramble siRNA (#sc-37007; Santa Cruz Biotechnology, Santa Cruz, CA, USA) or FGFR4 siRNA (#sc-35368; Santa Cruz Biotechnology, Santa Cruz, CA, USA). Transfection was started by using the Lipofectamine RNAiMAX Transfection Reagent (#13778030; Thermo Fisher Scientific, Cleveland, OH, USA) in Opti-MEM (#11058021; Gibco, Grand Island, NY, USA) for 12 h at first, then the medium was changed to complete DMEM for another 12 h to become ready for further experiments.

### 2.4. Histological Analysis and Immunohistochemistry

Hematoxylin-eosin and Sirius red staining was performed to assess heart tissue morphology and fibrosis. Mouse heart specimens were fixed in 4% paraformaldehyde solution, following standard dehydration protocols. The tissues were embedded in paraffin wax and subsequently sectioned at a thickness of 5 μm. Next, the specimens were deparaffinized, hydrated and stained by using commercial hematoxylin-eosin (#G1120; Solarbio, Beijing, China) and Sirius red (#G1470; Solarbio, Beijing, China) staining kits, according to the manufacturer’s instructions.

Hematoxylin-eosin staining was only used to observe the architectural changes of the mouse heart tissues. Fibrotic areas were quantified by Sirius red staining, to ensure representative sampling, at least 10 randomly selected non-overlapping fields per section were captured using a Nikon ECLIPSE Ni microscope (Nikon, Tokyo, Japan). The interstitial, together with perivascular collagen volume fraction (fibrosis area), was calculated as the ratio of the Sirius Red-positive area (red-stained collagen fibers) to the total tissue area, excluding perivascular spaces, using ImageJ software (version 1.46r). All image analyses were performed by two independent investigators blinded to the experimental groups to minimize observer bias.

For immunohistochemical evaluations, the 5 μm paraffin-embedded cardiac sections were initially deparaffinized and rehydrated. Endogenous peroxidase activity was effectively quenched by immersing the slides in a 3% hydrogen peroxide solution for a duration of 5 min. To facilitate optimal antigen unmasking, the sections were heated in a 0.01 M sodium citrate buffer solution (pH 6.0) at 95 °C for 10 min. After permeabilization with 0.5% Triton X-100 for 20 min, non-specific protein binding sites were blocked by incubation in a 5% bovine serum albumin (BSA) solution for 40 min at ambient room temperature. The slides were incubated with primary antibody against α-SMA (#19245, 1:500 dilution; Cell Signaling Technology, Danvers, MA, USA), or collagen 1A1 (#72026, 1:300 dilution; Cell Signaling Technology, Danvers, MA, USA) at 4 °C overnight. Sections were then incubated with appropriate secondary antibody and visualized by using a commercial DAB horseradish peroxidase color development kit (#P0202; Beyotime, Shanghai, China), followed by counterstaining with hematoxylin. Images were captured using a Nikon ECLIPSE Ni microscope (Nikon, Tokyo, Japan).

To ensure the accuracy and reproducibility of the quantification, all images were captured under identical illumination, magnification, and exposure settings. For each sample, at least 10 representative, non-overlapping fields of view were randomly selected. Quantitative analysis was performed using ImageJ software. The color deconvolution plugin was utilized to separate the hematoxylin (counterstain) and DAB (target protein) signals.

### 2.5. Immunoblotting

Total cellular and tissue proteins were thoroughly extracted utilizing RIPA lysis buffer (#20-188; Merck, Darmstadt, Germany) dynamically supplemented with a comprehensive protease and phosphatase inhibitor cocktail (#ab201119; Abcam, Cambridge, MA, USA) to prevent enzymatic degradation. The precise protein concentrations within the lysates were determined employing a highly sensitive BCA protein assay kit (#ab102536; Abcam, Cambridge, MA, USA). Following thermal denaturation at 95 °C for 5 min, equivalent amounts of protein lysates were subjected to 10% sodium dodecyl sulfate-polyacrylamide gels (SDS-PAGE). The separated proteins were subsequently electro-transferred onto Immobilon-P PVDF membranes (#IPVH00010; Merck, Darmstadt, Germany). To prevent non-specific antibody adherence, the membranes were blocked utilizing a 5% non-fat dry milk solution at room temperature for a period of 1 h. This was followed by an overnight incubation at 4 °C with the designated primary antibodies. Subsequently, the membranes were probed with corresponding horseradish peroxidase-conjugated secondary antibodies for 2 h at ambient temperature. Immunoreactive protein bands were visualized using the enhanced Amersham Imager 600 detection system (GE Healthcare Life Sciences, Piscataway, NJ, USA). The relative expression levels of β-actin were monitored universally to serve as the internal loading control, and exact densitometric quantification of the protein signals was performed using NIH ImageJ software (version 1.46r). Antibodies used in this study were FGF23 (#ab307420, 1:1000 dilution; Abcam, Cambridge, MA, USA), FGFR4 (#8562, 1:1000 dilution; Cell Signaling Technology, Danvers, MA, USA), α-SMA (#19245, 1:2000 dilution; Cell Signaling Technology, Danvers, MA, USA), collagen 1A1 (#72026, 1:1000 dilution; Cell Signaling Technology, Danvers, MA, USA), Cyclin D1 (#55506, 1:1000 dilution; Cell Signaling Technology, Danvers, MA, USA), Cyclin E1 (#20808, 1:1000 dilution; Cell Signaling Technology, Danvers, MA, USA), PCNA (#13110, 1:1000 dilution; Cell Signaling Technology, Danvers, MA, USA), p-ERK1/2 (#9101, 1:1000 dilution; Cell Signaling Technology, Danvers, MA, USA), ERK1/2 (#4696, 1:2000 dilution; Cell Signaling Technology, Danvers, MA, USA), and β-actin (#AC038, 1:10,000 dilution; ABclonal, Wuhan, China). The original images of immunoblotting can be found in Supplementary Figures S3–S7.

### 2.6. Quantitative PCR

The complete pool of total RNA was isolated from both murine cardiac tissues and cultured cellular samples employing the TRIzol reagent extraction method (#15596026; Invitrogen, Carlsbad, CA, USA). RNA concentration and optimal purity metrics were strictly verified using a NanoDrop 2000 spectrophotometer (Thermo Fisher Scientific, Cleveland, OH, USA). The isolated RNA templates were subsequently reverse transcribed to generate complementary DNA (cDNA) libraries using the robust GoScript reverse transcription system (#A5000; Promega, Madison, WA, USA). Quantitative real-time polymerase chain reaction (qPCR) amplification protocols were executed utilizing the highly sensitive ChamQ SYBR qPCR master mix (#Q311-02; Vazyme, Nanjing, China). The relative mRNA transcript abundance for each target gene was meticulously normalized against the endogenous reference gene, β-actin. The primer sequences of target genes used in this study are mouse *Fgf23* (F: ATGCTAGGGACCTGCCTTAGA, R: GGAGCCAAGCAATGGGGAA), mouse *Fgfr4* (F: TCCATGACCGTCGTACACAAT, R: ATTTGACAGTATTCCCGGCAG), mouse *Acta2* (F: CCCAGACATCAGGGAGTAATGG, R: TCTATCGGATACTTCAGCGTCA), mouse *Collagen 1a1* (F: GCTCCTCTTAGGGGCCACT, R: ATTGGGGACCCTTAGGCCAT), mouse *Collagen 3a1* (F: CTGTAACATGGAAACTGGGGAAA, R: CCATAGCTGAACTGAAAACCACC), mouse *Cyclin d1* (F: GCGTACCCTGACACCAATCTC, R: ACTTGAAGTAAGATACGGAGGGC), mouse *Cyclin e1* (F: CTCCGACCTTTCAGTCCGC, R: CACAGTCTTGTCAATCTTGGCA), mouse *Pcna* (F: TTGCACGTATATGCCGAGACC, R: GGTGAACAGGCTCATTCATCTCT), mouse *Mki67* (F: ATCATTGACCGCTCCTTTAGGT, R: GCTCGCCTTGATGGTTCCT), mouse *Actb* (F: GTGACGTTGACATCCGTAAAGA, R: GCCGGACTCATCGTACTCC), human *CYCLIN D1* (F: GCTGCGAAGTGGAAACCATC, R: CCTCCTTCTGCACACATTTGAA), human *MKI67* (F: ACGCCTGGTTACTATCAAAAGG, R: CAGACCCATTTACTTGTGTTGGA), and human *ACTB* (F: CATGTACGTTGCTATCCAGGC, R: CTCCTTAATGTCACGCACGAT).

### 2.7. Immunofluorescence Staining

To facilitate in vivo morphological tracking, freshly harvested cardiac specimens were submerged in a 4% paraformaldehyde fixing solution overnight at 4 °C. Subsequently, the tissues were sequentially subjected to cryoprotection by dehydration in 20% and 30% sucrose solutions overnight at 4 °C, respectively. The fully cryoprotected tissues were snap-frozen using Tissue-Tek O.C.T. compound (SAKURA, Tokyo, Japan), immersed in liquid nitrogen, and subsequently sliced into precise 10 μm cryosections. These sections were permeabilized using 0.5% Triton X-100 for a duration of 20 min, followed by a blocking step utilizing a 5% BSA solution for 40 min at room temperature. The slides were incubated with primary antibodies against FGF23 (#ab307420, 1:300 dilution; Abcam, Cambridge, MA, USA), α-SMA (#19245, 1:200 dilution; Cell Signaling Technology, Danvers, MA, USA), or collagen 1A1 (#72026, 1:200 dilution; Cell Signaling Technology, Danvers, MA, USA) at 4 °C overnight. Sections were then incubated with appropriate fluorescent secondary antibodies conjugated with Alexa Fluor 488 or Alexa Fluor 647. Finally, cell nuclei were stained with DAPI staining solution (#C1005; Beyotime, Shanghai, China). Images were captured using a Leica SP8 confocal microscope (Leica, Nussloch, Germany).

For in vitro studies, human primary cardiac fibroblasts were fixed by 4% paraformaldehyde solution for 15 min. This was followed by cellular permeabilization with 0.5% Triton X-100 for 20 min and subsequent blocking in a 5% BSA solution for 40 min at room temperature. Cells were incubated with primary antibodies against α-SMA (#19245, 1:200 dilution; Cell Signaling Technology, Beverly, MA, USA) or Ki67 (#9129, 1:400 dilution; Cell Signaling Technology, Beverly, MA, USA) at 4 °C overnight. Cells were then incubated with appropriate fluorescent secondary antibodies conjugated with Alexa Fluor 488 or Alexa Fluor 647. Finally, cell nuclei were stained with DAPI staining solution (#C1005; Beyotime, Shanghai, China). Images were captured using a Leica SP8 confocal microscope (Leica, Nussloch, Germany).

To ensure the reproducibility and objectivity of the quantification, all images were captured using identical exposure time, gain, and laser intensity settings across all experimental groups. At least 10 randomly selected, non-overlapping fields of view were imaged. Quantitative analysis was performed using ImageJ software. The mean fluorescence intensity or the percentage of positive area was calculated by setting a consistent threshold for each channel to distinguish specific signals from background noise. Interstitial together with perivascular positive staining were all calculated.

### 2.8. RNA Sequencing

To profile gene expression differences, total RNA from fresh mouse heart tissues was extracted and then used to construct cDNA libraries. Deep sequencing of these double-ended libraries was subsequently executed on the advanced BGISEQ 500 genomic platform, yielding a highly accurate read length of 50 base pairs (bp). The generated raw sequencing reads were computationally aligned to the Ensembl murine reference genome assembly (mm10/grcm38) leveraging HISAT2 mapping software (version 2.1.0). To facilitate downstream processing, SAMtools algorithms were utilized to efficiently convert the generated sequence alignments into the standardized Binary Alignment Map (BAM) format. The relative transcript abundance, strictly defined as fragments per kilobase of transcript per million mapped reads (FPKM), was rigorously calculated utilizing StringTie software (version 2.2.1). To ensure robust statistical modeling, the raw count matrix was appropriately normalized employing the DESeq2 package (version 1.48.2).

RNA sequencing was performed by Genedenovo Biotechnology Co., Ltd. (Guangzhou, China), and the bioinformatics analysis was performed by the OmicShare online platform of Genedenovo Biotechnology Co., Ltd. All differentially expressed genes (DEGs) reported in the RNA-seq analysis were filtered using a stringent False Discovery Rate (FDR) threshold (adjusted *p* < 0.05) to maintain statistical rigor.

### 2.9. Cell Proliferation Assay

MTT Cell Proliferation and Cytotoxicity Assay Kit (#C0009S; Beyotime, Shanghai, China) and Cell Counting Kit-8 (#C0037; Beyotime, Shanghai, China) were used to test the proliferation capacity of human primary cardiac fibroblasts according to the manufacturer’s instructions.

### 2.10. Statistical Analysis

Values in this study were expressed as mean ± standard deviation (SD). Statistical analysis was performed using GraphPad Prism 9 software. Sample sizes were chosen based on preliminary experiments and power analysis to ensure adequate power to detect the observed effects. Differences between the two groups were analyzed by using a two-tailed, unpaired Student’s *t*-test. Differences between more than two groups were analyzed by using one-way analysis of variance (ANOVA) followed by Dunnett’s multiple comparisons test. All data presented in this study followed a normal distribution and showed homogeneity of variance. A *p*-value < 0.05 was considered statistically significant.

## 3. Results

### 3.1. FGF23 Is Significantly Upregulated in Mouse Pressure-Overloaded Myocardium

**Figure 1:**
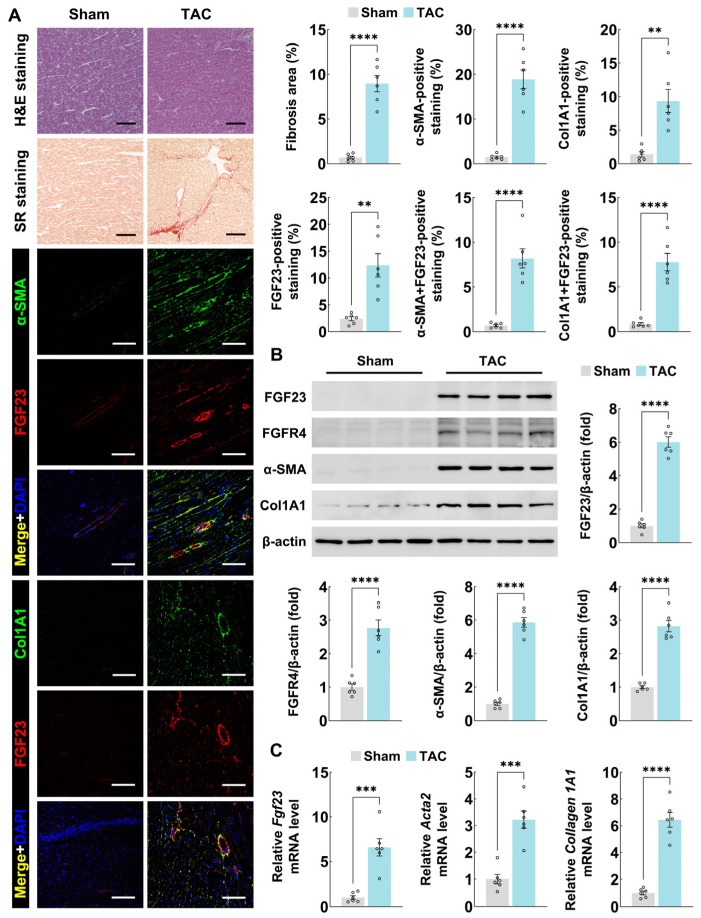
FGF23 is significantly upregulated in mouse pressure-overloaded myocardium. (**A**) Hematoxylin-eosin staining, Sirius red staining, and immunofluorescence staining of α-SMA/FGF23 and collagen 1A1/FGF23 expression in cardiac sections from C57BL/6 mice after sham or TAC operation for 8 weeks (n = 6 mice per group, scale bar = 100 μm), (**B**) immunoblotting analyses of FGF23, FGFR4, α-SMA, and collagen 1A1 protein expression in heart tissues from C57BL/6 mice after sham or TAC operation for 8 weeks (n = 6 mice per group), and (**C**) quantitative PCR analyses of *Fgf23*, *Acta2* (α-SMA), and *Collagen 1A1* mRNA expression in heart tissues from C57BL/6 mice after sham or TAC operation for 8 weeks (n = 6 mice per group). H&E, hematoxylin-eosin; SR, Sirius red; and Col1A1, collagen 1A1. Data are displayed as mean ± SD. Each dot represents an individual animal. *p*-value analyzed by two-tailed Student’s *t*-test (**A**–**C**). ** *p* < 0.01, *** *p* < 0.001, and **** *p* < 0.0001.

To investigate the role of FGF23 in cardiac fibrosis, we first examined its expression in the myocardium of mice subjected to pressure overload via TAC. As shown in Figure 1A, Hematoxylin-eosin and Sirius red staining revealed extensive tissue fibrosis in TAC hearts compared to Sham controls. Meanwhile, immunofluorescence staining showed that FGF23 was upregulated along with the expression of α-SMA and collagen 1A1 (Figure 1A), indicating that FGF23 may play a role in the development of cardiac fibrosis.

Importantly, by using immunoblotting and quantitative PCR, we found that both protein and mRNA levels of FGF23 were markedly elevated in TAC-stressed mice hearts, along with the upregulation of FGFR4 (the specific receptor of FGF23), α-SMA, and collagen 1A1 (Figure 1B,C). These data establish a correlation between pressure overload-induced myocardial fibrosis and upregulated cardiac FGF23 expression.

### 3.2. FGF23 Monoclonal Antibody Treatment Attenuates Cardiac Fibrosis Progression in Mice

**Figure 2:**
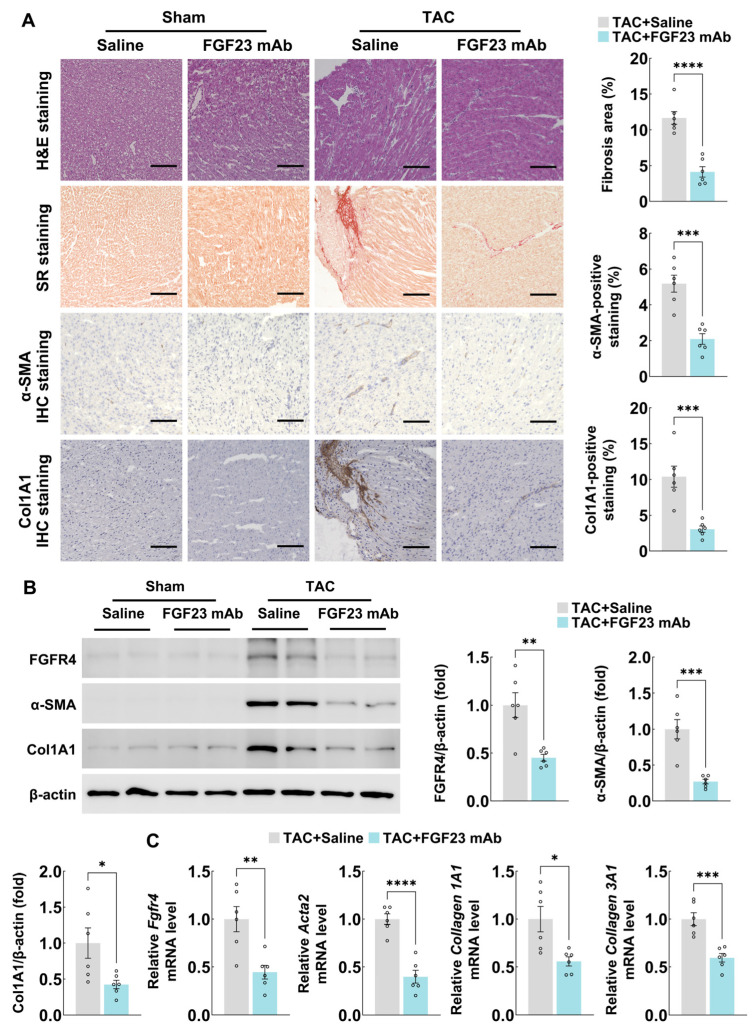
FGF23 monoclonal antibody treatment attenuates cardiac fibrosis progression in mice. (**A**) Hematoxylin-eosin staining, Sirius red staining, and immunohistochemistry staining of α-SMA/ and collagen 1A1 expression in cardiac sections from C57BL/6 mice after sham or TAC operation (with or without FGF23 mAb treatment) for 8 weeks (n = 6 mice per group, scale bar = 100 μm), (**B**) immunoblotting analyses of FGFR4, α-SMA, and collagen 1A1 protein expression in heart tissues from C57BL/6 mice after sham or TAC operation (with or without FGF23 mAb treatment) for 8 weeks (n = 6 mice per group), and (**C**) quantitative PCR analyses of *Fgfr4*, *Acta2* (α-SMA), *Collagen 1A1*, and *Collagen 3A1* mRNA expression in heart tissues from C57BL/6 mice after sham or TAC operation (with or without FGF23 mAb treatment) for 8 weeks (n = 6 mice per group). H&E, hematoxylin-eosin; SR, Sirius red; and Col1A1, collagen 1A1. Data are displayed as mean ± SD. Each dot represents an individual animal. *p*-value analyzed by two-tailed Student’s *t*-test (**A**–**C**). * *p* < 0.05, ** *p* < 0.01, *** *p* < 0.001, and **** *p* < 0.0001.

To determine whether FGF23 contributes to cardiac fibrosis, we investigated the effects of inhibiting FGF23 activity using a specific monoclonal antibody. Mice subjected to TAC were treated with either an FGF23 mAb or an isotype-matched IgG control for eight weeks. Histological analysis with hematoxylin-eosin staining and Sirius red staining showed that FGF23 inhibition by monoclonal antibody significantly ameliorated myocardial fibrosis in TAC mouse models (Figure 2A). Consistently, immunohistochemistry of α-SMA and collagen 1A1 showed the same result (Figure 2A).

Immunoblotting and quantitative PCR results showed that FGF23 mAb treatment also inhibited FGFR4 expression in fibrotic mouse hearts, which suggests that FGF23 may function through FGFR4 during pressure overload (Figure 2B,C). Moreover, the protein and mRNA expression of fibrotic markers, including α-SMA, collagen 1A1, and collagen 3A1, were all significantly downregulated in the heart tissues of FGF23 mAb-treated TAC group (Figure 2B,C). These results indicate that blocking FGF23 signaling can alleviate pressure overload-induced cardiac fibrosis.

### 3.3. Recombinant FGF23 Administration Aggravates Myocardial Fibrosis in Pressure-Overloaded Mouse Hearts

**Figure 3:**
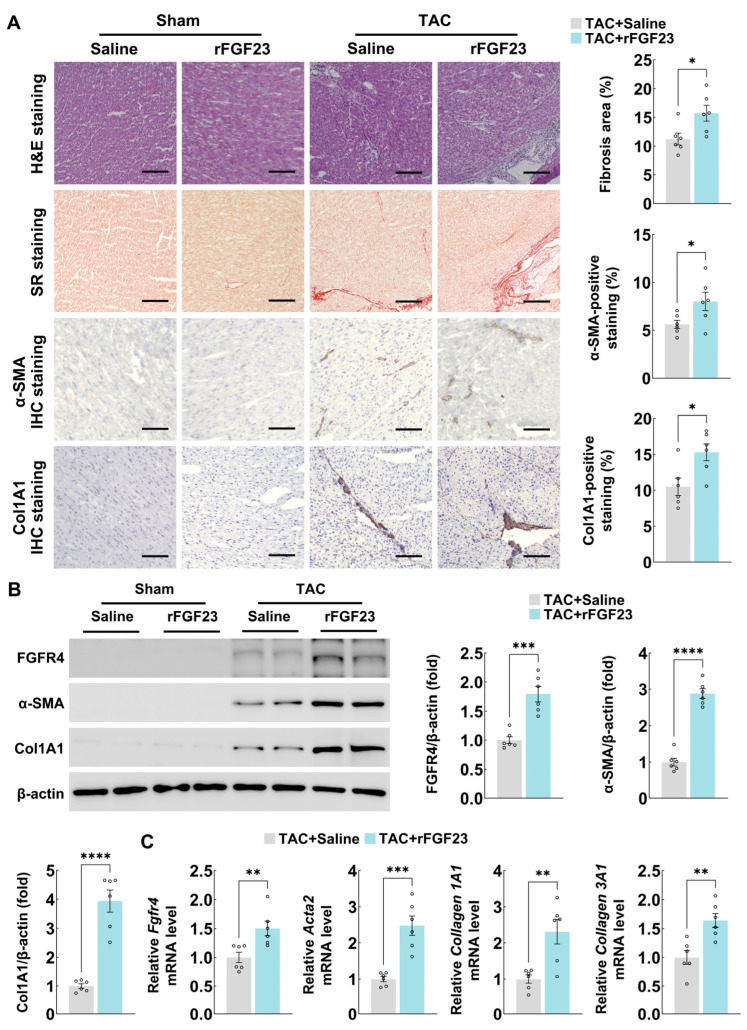
Recombinant FGF23 administration aggravates myocardial fibrosis in pressure-overloaded mouse hearts. (**A**) Hematoxylin-eosin staining, Sirius red staining, and immunohistochemistry staining of α-SMA/ and collagen 1A1 expression in cardiac sections from C57BL/6 mice after sham or TAC operation (with or without rFGF23 treatment) for 8 weeks (n = 6 mice per group, scale bar = 100 μm), (**B**) immunoblotting analyses of FGFR4, α-SMA, and collagen 1A1 protein expression in heart tissues from C57BL/6 mice after sham or TAC operation (with or without rFGF23 treatment) for 8 weeks (n = 6 mice per group), and (**C**) quantitative PCR analyses of *Fgfr4*, *Acta2* (α-SMA), *Collagen 1A1*, and *Collagen 3A1* mRNA expression in heart tissues from C57BL/6 mice after sham or TAC operation (with or without rFGF23 treatment) for 8 weeks (n = 6 mice per group). H&E, hematoxylin-eosin; SR, Sirius red; and Col1A1, collagen 1A1. Data are displayed as mean ± SD. Each dot represents an individual animal. *p*-value analyzed by two-tailed Student’s *t*-test (**A**–**C**). * *p* < 0.05, ** *p* < 0.01, *** *p* < 0.001, and **** *p* < 0.0001.

Conversely, we examined the effect of supplementing FGF23 by administering rFGF23 to TAC mice. Histological analysis with hematoxylin-eosin staining and Sirius red staining revealed a substantial increase in tissue fibrosis in TAC mouse hearts treated with rFGF23 compared to vehicle-treated TAC hearts (Figure 3A). Accordingly, immunohistochemistry indicated that FGF23 overexpression further promoted the expression of α-SMA and collagen 1A1 in mouse hearts after TAC surgery (Figure 3A).

Similarly, by using immunoblotting and quantitative PCR, we found the expression of FGFR4 was further upregulated in mouse heart tissues by rFGF23 supplementation after TAC, along with the additional increase in fibrotic markers α-SMA, collagen 1A1, and collagen 3A1 (Figure 3B,C). These gain-of-function data reinforce the notion that FGF23 promotes the progression of myocardial fibrosis under pressure overload.

### 3.4. FGF23 Expression Is Associated with Fibroblast Proliferation and Activation in Myocardial Fibrosis

**Figure 4:**
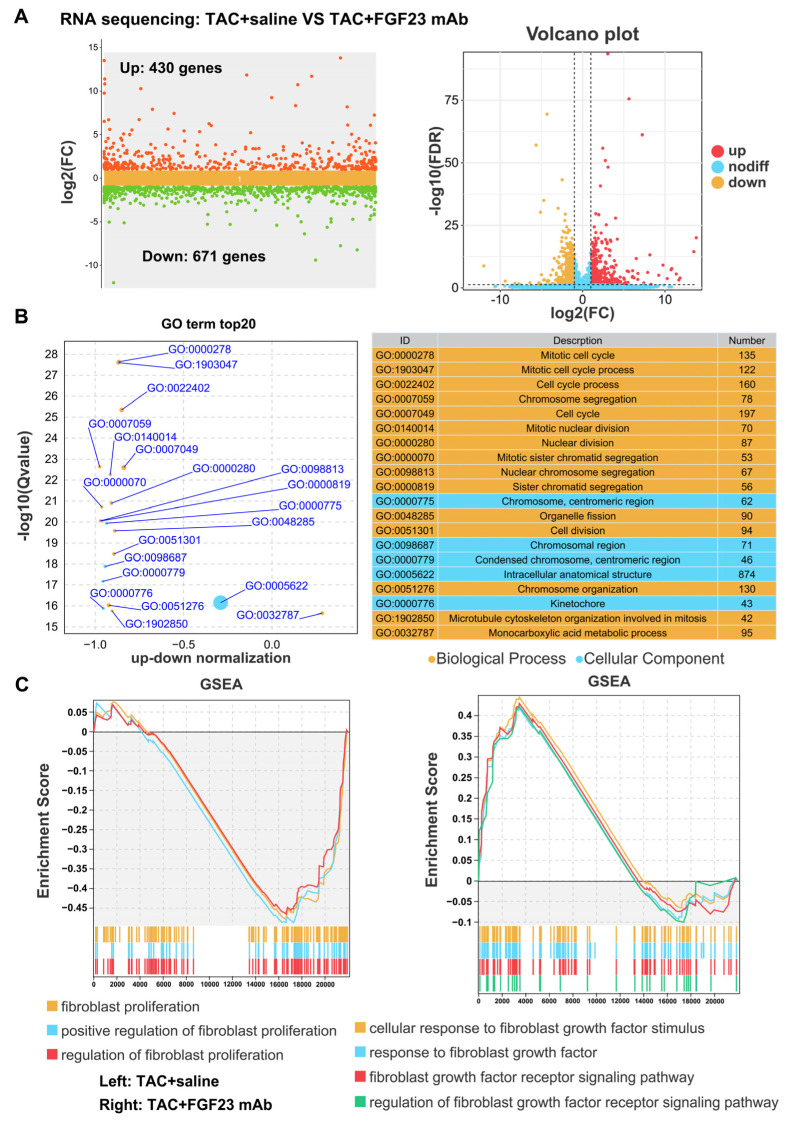
FGF23 expression is associated with fibroblast proliferation and activation in myocardial fibrosis. (**A**) RNA sequencing analysis of differentially expressed genes in heart tissues from C57BL/6 mice after TAC operation (with or without FGF23 mAb treatment) for 8 weeks (n = 3 mice per group), left: scatter plots of multiple group differences, right: volcano plots, (**B**) gene ontology (GO) analysis of significantly changed biological processes and cellular components based on the RNA sequencing data of C57BL/6 mice after TAC operation (with or without FGF23 mAb treatment) for 8 weeks (n = 3 mice per group), and (**C**) gene set enrichment analysis (GSEA) of significantly changed signaling pathways based on the RNA sequencing data of C57BL/6 mice after TAC operation (with or without FGF23 mAb treatment) for 8 weeks (n = 3 mice per group).

To explore the mechanisms underlying the pro-fibrotic effect of FGF23 under pressure overload, we performed RNA sequencing on left ventricular tissues from TAC-operated mice treated with or without FGF23 mAb (Figure 4A). Gene ontology (GO) analysis of differentially expressed genes revealed that FGF23 signaling inhibition significantly affected cell cycle and cell division processes in TAC mouse hearts (Figure 4B). As the proliferation and activation of cardiac fibroblasts commonly contribute to the development of myocardial fibrosis [20,21,22], we speculate that FGF23 may affect TAC-induced cardiac fibrosis via regulating cardiac fibroblast proliferation.

Based on the RNA sequencing data, we performed gene set enrichment analysis (GSEA). GSEA indicated a strong correlation between the TAC-induced gene signature and gene sets involved in “fibroblast proliferation”, “FGF stimulus”, “FGF response”, and “FGFR signaling” (Figure 4C), which showed that FGF23 and the associated FGFR signaling inhibition alleviated fibroblast proliferation and activation in pressure overload.

### 3.5. FGF23 Regulates MAPK/ERK Signaling Activation and Proliferation-Related Marker Gene Expression After TAC Operation

**Figure 5:**
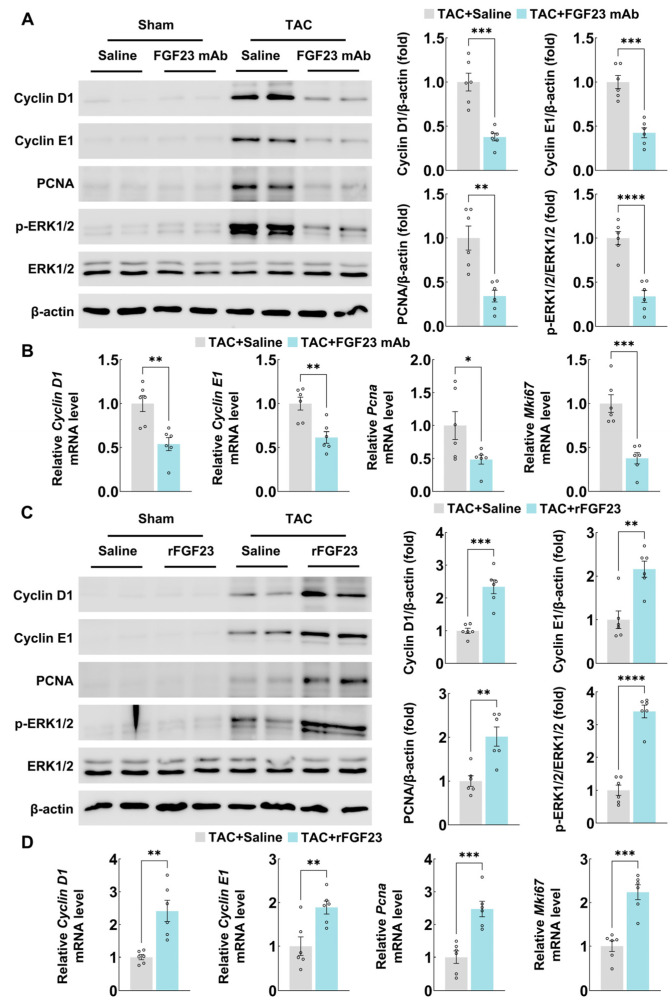
FGF23 regulates MAPK/ERK signaling activation and proliferation-related marker gene expression after TAC operation. (**A**) Immunoblotting analyses of Cyclin D1, Cyclin E1, PCNA, phosphorylated ERK1/2, and ERK1/2 protein expression in heart tissues from C57BL/6 mice after sham or TAC operation (with or without FGF23 mAb treatment) for 8 weeks (n = 6 mice per group), (**B**) quantitative PCR analyses of *Cyclin D1*, *Cyclin E1*, *Pcna*, and *Mki67* mRNA expression in heart tissues from C57BL/6 mice after sham or TAC operation (with or without FGF23 mAb treatment) for 8 weeks (n = 6 mice per group), (**C**) immunoblotting analyses of Cyclin D1, Cyclin E1, PCNA, phosphorylated ERK1/2, and ERK1/2 protein expression in heart tissues from C57BL/6 mice after sham or TAC operation (with or without rFGF23 treatment) for 8 weeks (n = 6 mice per group), and (**D**) quantitative PCR analyses of *Cyclin D1*, *Cyclin E1*, *Pcna*, and *Mki67* mRNA expression in heart tissues from C57BL/6 mice after sham or TAC operation (with or without rFGF23 treatment) for 8 weeks (n = 6 mice per group). Data are displayed as mean ± SD. Each dot represents an individual animal. *p*-value analyzed by two-tailed Student’s *t*-test (**A**–**C**). * *p* < 0.05, ** *p* < 0.01, *** *p* < 0.001, and **** *p* < 0.0001.

To further support the data that FGF23 regulates cell proliferation in myocardial fibrosis, we performed immunoblotting analysis. Our data showed that proliferation-related markers, including Cyclin D1, Cyclin E1, and PCNA, were all upregulated after TAC surgery in mouse heart tissues, while FGF23 inhibition by monoclonal antibody downregulated the expression of proliferation-related proteins after TAC (Figure 5A). Additionally, quantitative PCR revealed the same result (Figure 5B).

In FGF23 overexpression mouse models, rFGF23 supplementation further upregulated Cyclin D1, Cyclin E1, and PCNA expression after TAC surgery (Figure 5C). Meanwhile, the mRNA levels of Cyclin D1, Cyclin E1, PCNA, and Ki67 were all significantly changed by rFGF23 (Figure 5D). These data showed that FGF23 can regulate proliferation-related marker gene expression after TAC operation.

The MAPK/ERK signaling pathway is the main downstream of FGFs/FGFRs, which also significantly contributes to cell proliferation in various tissues and organs [23,24,25,26]. We therefore checked the change of MAPK/ERK signaling in our mouse models. As shown in Figure 5A,C, immunoblotting analysis of heart tissues from TAC mice revealed increased phosphorylation of ERK1/2 (p-ERK1/2) compared to sham controls. Furthermore, treatment with FGF23 mAb in TAC mice significantly reduced p-ERK1/2 levels, while rFGF23 administration further enhanced p-ERK1/2 activation. This indicates that FGF23 signaling is linked to the activation of the MAPK/ERK pathway in the pressure-overloaded mouse heart.

### 3.6. FGF23/FGFR4 Signaling Regulates Human Primary Cardiac Fibroblasts Proliferation and Activation In Vitro

**Figure 6:**
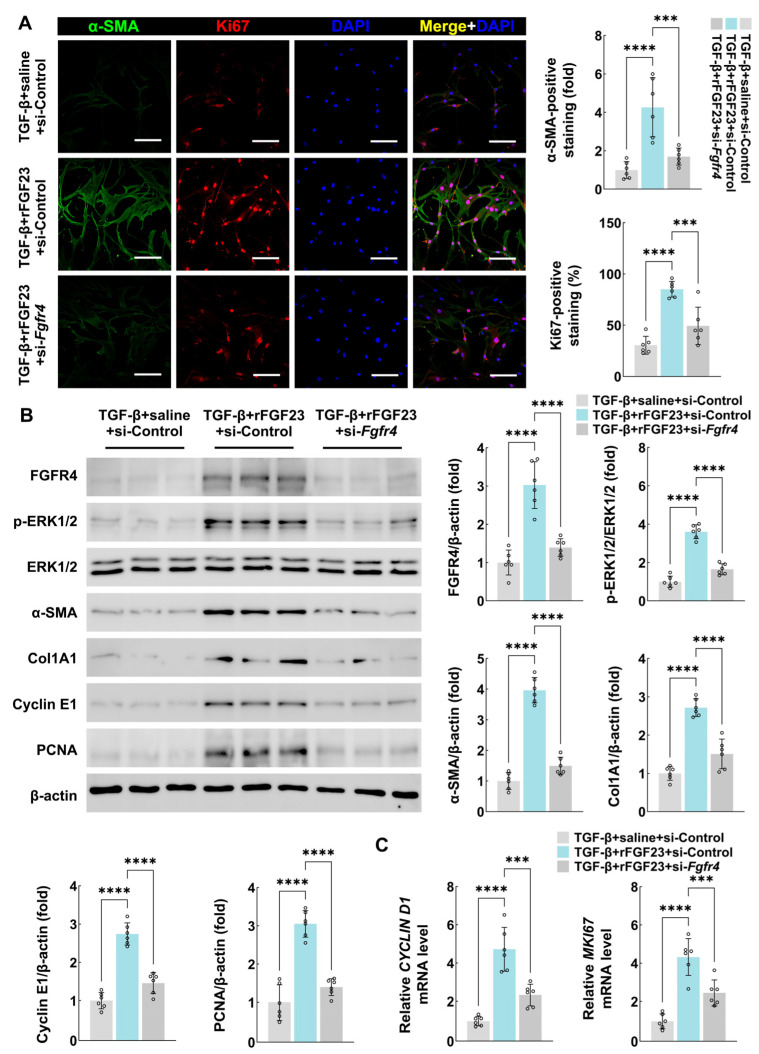
FGF23/FGFR4 signaling regulates human primary cardiac fibroblast proliferation and activation in vitro. (**A**) Immunofluorescence staining of α-SMA and Ki67 expression in human primary cardiac fibroblasts treated with TGF-β/rFGF23 (with or without *Fgfr4* knockdown) (n = 6 biologically independent experiments, scale bar = 100 μm), (**B**) immunoblotting analyses of FGFR4, phosphorylated ERK1/2, ERK1/2, α-SMA, collagen 1A1, Cyclin E1, and PCNA protein expression in human primary cardiac fibroblasts treated with TGF-β/rFGF23 (with or without *Fgfr4* knockdown) (n = 6 biologically independent experiments), and (**C**) quantitative PCR analyses of *CYCLIN D1* and *MKI67* mRNA expression in human primary cardiac fibroblasts treated with TGF-β/rFGF23 (with or without *Fgfr4* knockdown) (n = 6 biologically independent experiments). Col1A1 denotes collagen 1A1. Data are displayed as mean ± SD. Each dot represents a biologically independent experiment. *p*-value analyzed by ordinary one-way ANOVA with Dunnett’s multiple comparisons test (**A**–**C**). *** *p* < 0.001, and **** *p* < 0.0001.

To elucidate the direct effects of FGF23 on cardiac fibroblasts, we treated human primary cardiac fibroblasts with rFGF23 under TGF-β-stimulated conditions, as TGF-β is a known inducer of fibroblast activation [27,28]. The in vitro experiments demonstrated that rFGF23 treatment significantly increased cardiac fibroblast proliferation and activation under TGF-β stimulation, as indicated by α-SMA/Ki67 immunofluorescence staining, α-SMA/collagen 1A1/Cyclin E1/PCNA immunoblotting, and Cyclin D1/Ki67 quantitative PCR (Figure 6A–C).

Furthermore, we investigated the role of FGFR4 in human primary cardiac fibroblasts. Small interfering RNA-mediated FGFR4 knockdown abrogated rFGF23-induced proliferation and activation of cardiac fibroblasts, as well as the activation of the MAPK/ERK signaling pathway (Figure 6A–C). Interestingly, we found that FGF23 alone cannot induce fibroblast activation or proliferation in vitro (Supplementary Figure S1). Meanwhile, MTT and CCK8 experiments also confirmed the above results (Supplementary Figure S2). Overall, these findings strongly suggest that FGF23 exerts its pro-fibrotic effects on cardiac fibroblasts through its receptor FGFR4.

## 4. Discussion

Myocardial fibrosis is a critical pathological process that underlies the progression of heart failure, leading to impaired cardiac function and increased mortality [29]. Identifying molecular players that drive fibrosis is essential for developing effective therapeutic strategies. In this study, we demonstrate that FGF23, a hormone traditionally known for its role in mineral metabolism [30], plays a significant pro-fibrotic role in the heart. Our findings reveal that FGF23 promotes myocardial fibrosis by enhancing cardiac fibroblast proliferation and activation, mediated through the FGFR4 receptor and downstream MAPK/ERK signaling pathway.

We observed a significant upregulation of FGF23 in the pressure-overloaded myocardium of mice subjected to TAC, a well-established model of cardiac hypertrophy and fibrosis [31,32]. Previous studies suggest that FGF23 is mainly secreted by the bone or the kidney. However, our study indicated the potential local production of FGF23 in cardiac tissue under pressure overload. This increase in FGF23 levels correlated with the development of cardiac fibrosis, suggesting a potential association between FGF23 and this pathological process. Consistent with this observation, inhibiting FGF23 activity with a monoclonal antibody significantly attenuated TAC-induced cardiac fibrosis and partially improved cardiac function. Conversely, administration of rFGF23 exacerbated myocardial fibrosis in TAC mice, further identifying the pro-fibrotic role of FGF23 in pressure overload.

Our investigation into the underlying mechanisms revealed that FGF23 directly influences cardiac fibroblasts, the key effector cells in fibrosis [33]. In vitro studies using human primary cardiac fibroblasts demonstrated that FGF23 treatment, along with TGF-β, significantly promoted fibroblast proliferation and upregulated the expression of key markers of fibroblast activation, like α-SMA. This suggests that FGF23 acts as a potent stimulus for fibroblast-mediated ECM deposition, as well as cell proliferation.

Our study also elucidated the signaling pathways involved in FGF23-mediated cardiac fibroblast activation. We found that FGF23 activates the MAPK/ERK signaling pathway. This pathway is known to play a crucial role in regulating cell growth, differentiation, and ECM production in cardiac fibroblasts [34,35]. Inhibition of FGFR4, the main receptor for FGF23, abrogated the pro-fibrotic effects of FGF23 on cardiac fibroblasts, including their proliferation, activation, and MAPK/ERK signaling activation. This indicates that FGF23 signals through FGFR4 to promote cardiac fibrosis. It should be noted that FGFR4 expression in cardiomyocytes may also contribute to the progression of myocardial fibrosis, however, our study mainly focused on the role of FGF23 in cardiac fibroblasts.

The findings of this study have some implications for the understanding and treatment of myocardial fibrosis. Elevated FGF23 levels have been associated with adverse cardiovascular outcomes, including HF, in multiple epidemiological studies [36,37,38]. This study suggests that FGFR4 serves as the primary receptor during myocardial fibrosis, while FGF23 usually requires α-Klotho as a co-receptor in the kidneys. Previous studies suggest FGF23 can act independently of Klotho in the heart via FGFR4 [12]. Our research provides a direct mechanistic link between FGF23 and cardiac fibrosis, suggesting that FGF23 is not only a biomarker but also an active contributor to the fibrotic process, especially under pressure overload. Therefore, targeting FGF23 and the associated signaling pathways represents mechanistic insights for mitigating myocardial fibrosis and improving outcomes in patients with heart failure.

By using FGF23 monoclonal antibody and recombinant protein, we found that FGF23 largely affected the expression of its main receptor, FGFR4, in cardiac tissues under pressure overload. These data suggested that FGFR4 regulation in myocardial fibrosis is expected to be ligand-dependent, and also indicated a different mechanism of FGFR4 signaling in the heart. Interestingly, a recent study suggests that FGF21 has the potential to control the expression of FGFR1 [39], which is consistent with our study and represents mechanistic insights between FGFs and FGFRs.

While this study provides evidence for FGF23’s role in cardiac fibrosis, several aspects still need to be further investigated. We used a well-established pressure overload mouse model in this study. However, the role of FGF23 in other etiologies of HF, such as myocardial infarction and diabetic cardiomyopathy, warrants investigation. We did not perform echocardiography experiments in our mouse models, which limits a better characterization of the functional impact of FGF23 in reducing cardiac fibrosis. Meanwhile, the potential effects of FGF23 on other cell types in the heart, such as macrophages or endothelial cells, which also influence myocardial fibrosis, were not explored. The effects of FGF23 on other cardiac cell populations in our mouse models were not examined. Additionally, clinical translation of FGF23 would require validation in human disease cohorts and interventional trials. It should also be noted that while our mechanistic findings are consistent and statistically significant, future studies with larger sample sizes or validation in different models would be beneficial to further strengthen the robustness of these observations.

## 5. Conclusions

In conclusion, our study establishes a role for FGF23 in the pathogenesis of myocardial fibrosis. We demonstrate that FGF23 is upregulated in pressure-overloaded mouse hearts and that its inhibition attenuates cardiac fibrosis. Mechanistically, FGF23 promotes cardiac fibroblast proliferation and activation via the receptor FGFR4 and the downstream MAPK/ERK signaling. These findings highlight that FGF23 can serve as a critical mediator of cardiac fibrosis, and targeting FGF23 holds mechanistic insight for treating heart failure and the associated myocardial fibrosis.

## Data Availability

Data not already included in the manuscript may be obtained from the corresponding authors on reasonable requests.

## Supplementary Materials

The following supporting information can be downloaded at https://www.mdpi.com/article/10.3390/biology15070539/s1, Figure S1: FGF23 alone did not affect human primary cardiac fibroblasts activation and proliferation; Figure S2: MTT and CCK8 assay demonstrating cell proliferation capacities in human primary cardiac fibroblasts; Figure S3: Original western blot images of Figure 1; Figure S4: Original western blot images of Figure 2; Figure S5: Original western blot images of Figure 3; Figure S6: Original western blot images of Figure 5; Figure S7: Original western blot images of Figure 6.

## Abbreviations

The following abbreviations are used in this manuscript:

HF	Heart failure
TAC	Transverse aortic constriction
FGF23	Fibroblast growth factor 23
rFGF23	Recombinant FGF23
ECM	Extracellular matrix
FGFR	Fibroblast growth factor receptor
α-SMA	α-smooth muscle actin
MAPK	Mitogen-activated protein kinase
DMEM	Dulbecco’s modification of eagle’s medium
BSA	Bovine serum albumin
Col1A1	Collagen 1A1
