# Supplementary material for: FGF23 Controls Myocardial Fibrosis Progression via Promoting Cardiac Fibroblast Proliferation and Activation in Mice
Source: Biology (Basel). 2026 Mar 27;15(7):539. doi: 10.3390/biology15070539 (PMC13072025; doi:10.3390/biology15070539)
Supplement: Supplementary file 1 [file biology-15-00539-s001.zip › biology-4171821-supplementary.pdf]

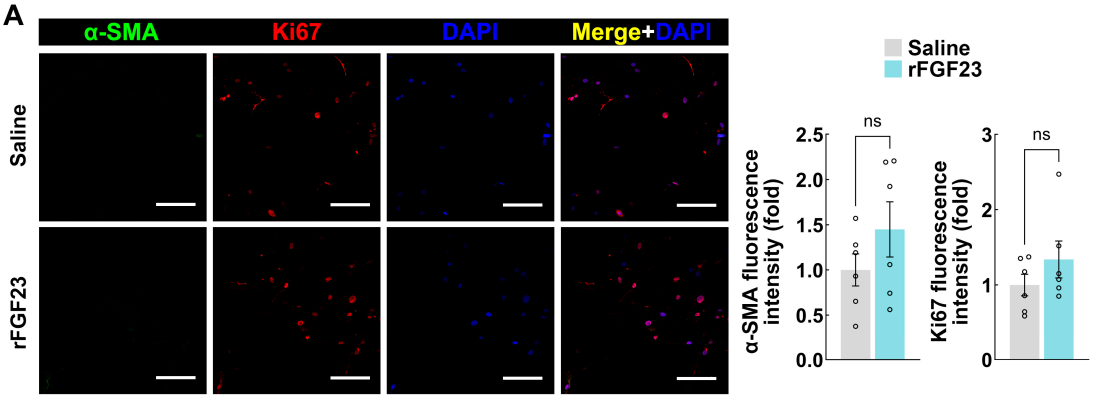


**Supplementary Figure S1.** FGF23 alone did not affect human primary cardiac fibroblasts activation and proliferation. (**a**) Immunofluorescence staining of *α*-SMA and Ki67 expression in human primary cardiac fibroblasts treated with saline or rFGF23 (n = 6 biologically independent experiments, scale bar = 100 μm). Data are displayed as mean ± SD. Each dot represents a biologically independent experiment. p value analyzed by two-tailed Student's t test (A). ns: non-significant.

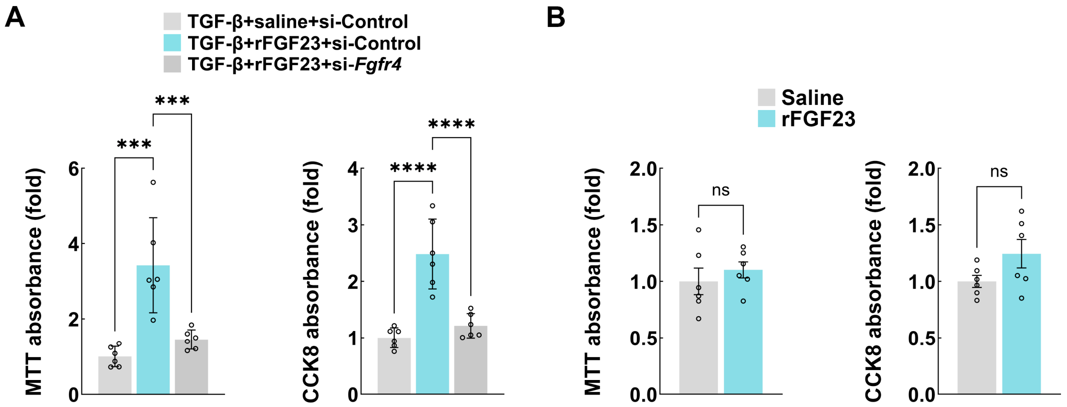


**Supplementary Figure S2.** MTT and CCK8 assay demonstrating cell proliferation capacities in human primary cardiac fibroblasts. (**a**) MTT and CCK8 absorbance of human primary cardiac fibroblasts treated with TGF-β/rFGF23 (with or without *Fgfr4* knockdown) (n = 6 biologically independent experiments); (**b**) MTT and CCK8 absorbance of human primary cardiac fibroblasts treated with saline or rFGF23 (n = 6 biologically independent experiments). Data are displayed as mean ± SD. Each dot represents a biologically independent experiment. p value analyzed by ordinary one-way ANOVA with Dunnett's multiple comparisons test (A) and two-tailed Student's t test (B). *$p < 0.05$, **$p < 0.01$, ***$p < 0.001$, ****$p < 0.0001$, ns: non-significant.

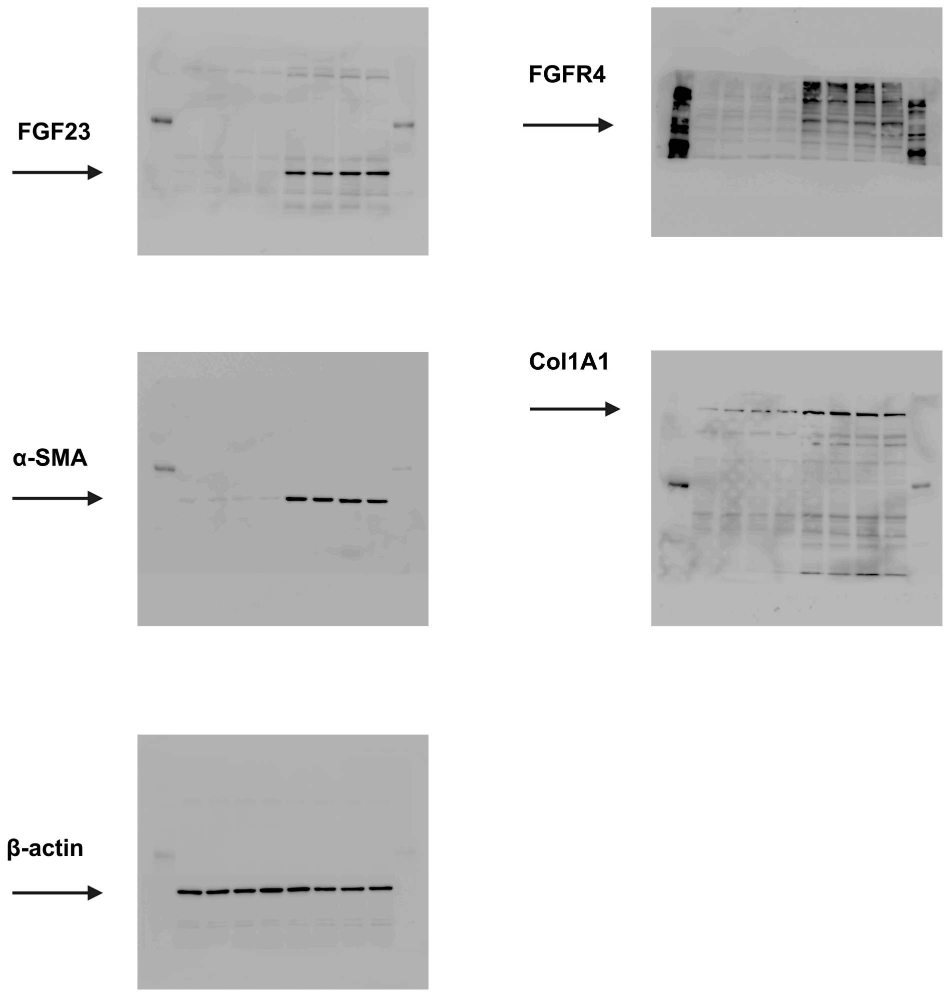


**Supplementary Figure S3.** Original western blot images of Figure 1.

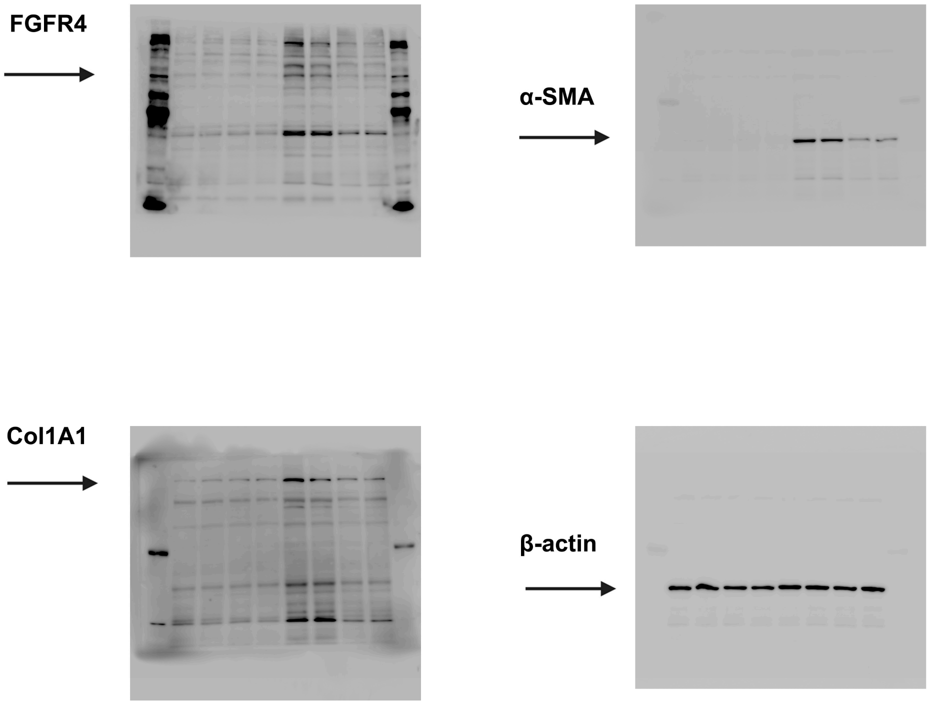


**Supplementary Figure S4.** Original western blot images of Figure 2.

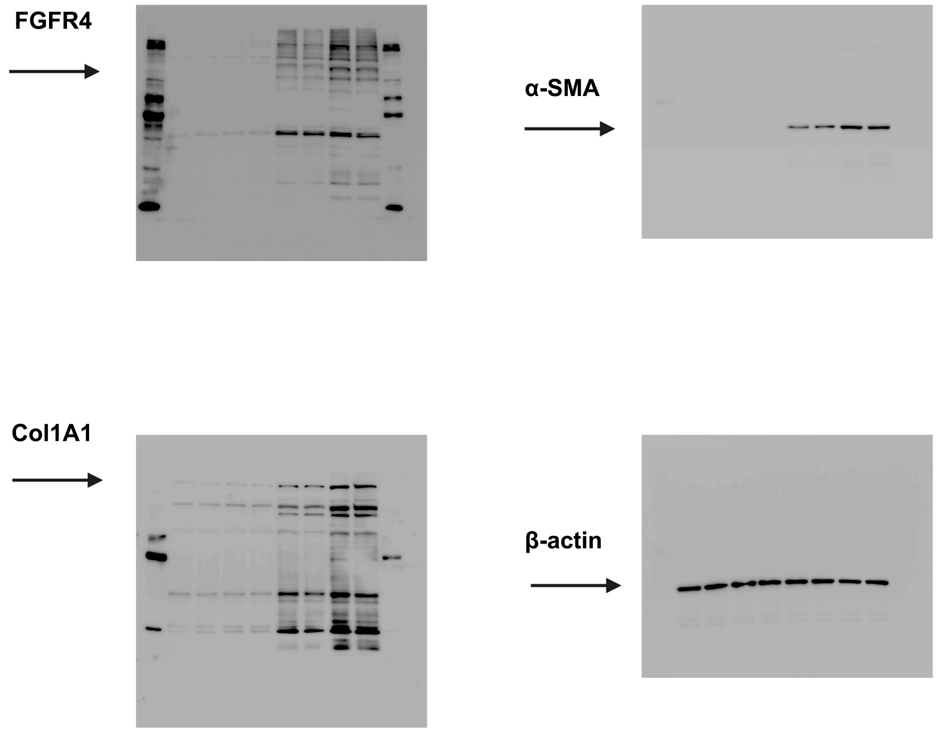


**Supplementary Figure S5.** Original western blot images of Figure 3.

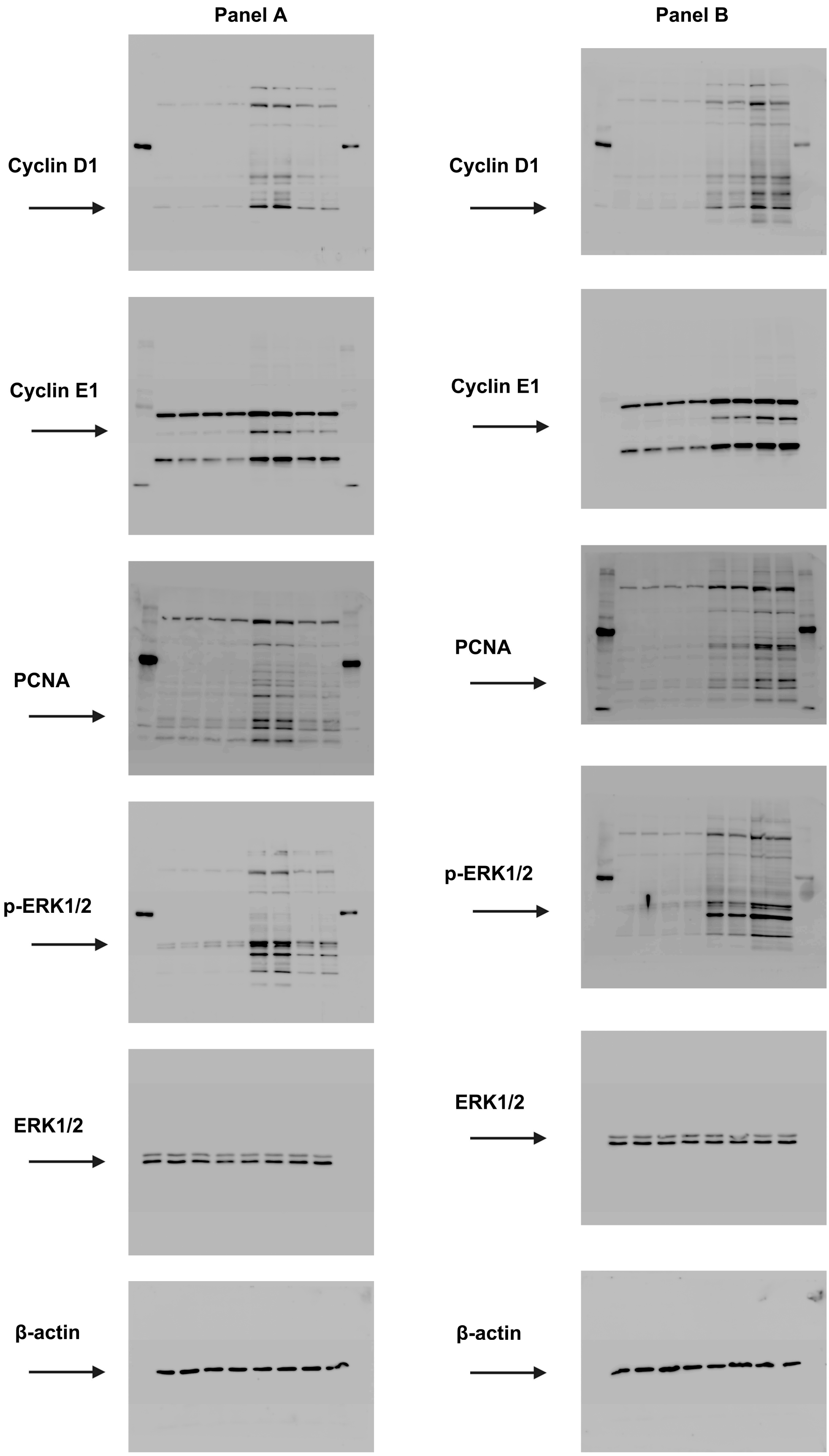


**Supplementary Figure S6.** Original western blot images of Figure 5.

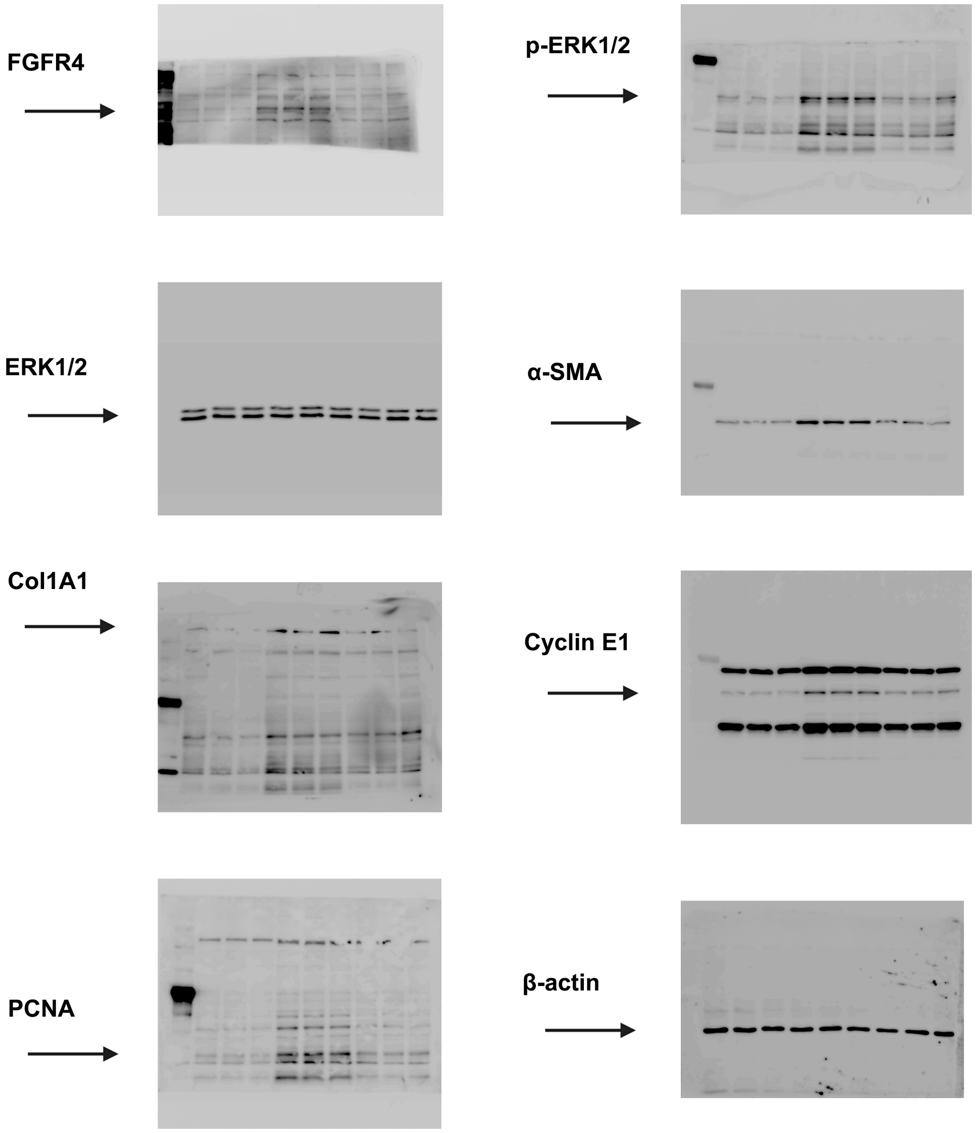


**Supplementary Figure S7.** Original western blot images of Figure 6.
